# Ten‐year minimal follow‐up of lateral opening wedge distal femoral osteotomy for lateral femorotibial osteoarthritis: Good survivorship and high patient satisfaction

**DOI:** 10.1002/ksa.12404

**Published:** 2024-08-06

**Authors:** Nicolas Cance, Cécile Batailler, Timothy Lording, Axel Schmidt, Sébastien Lustig, Elvire Servien

**Affiliations:** ^1^ Orthopedic Surgery Department Croix‐Rousse Hospital Lyon France; ^2^ Univ Lyon, Université Claude Bernard Lyon 1, IFSTTAR Villeurbanne France; ^3^ Melbourne Orthopaedic Group Windsor Victoria Australia; ^4^ IBM – EA 7424, Interuniversity Laboratory of Biology of Mobility Claude Bernard Lyon 1 University Lyon France

**Keywords:** conservative treatment, distal femoral osteotomy, genu valgum, knee arthroplasty, lateral tibiofemoral osteoarthritis, opening wedge

## Abstract

**Purpose:**

This study aimed (1) to determine complications and survival rates of lateral opening wedge distal femoral osteotomy (LOW‐DFO) in the long term, (2) to assess their clinical outcomes in the long term and (3) to identify risk factors of failure.

**Methods:**

Between 1991 and 2011, 62 LOW‐DFOs were performed in the same department. Inclusion criteria were all isolated LOW‐DFO performed for isolated lateral tibiofemoral osteoarthritis and valgus malalignment, with a minimum 10‐year follow‐up. Thirty‐eight patients were included, with a mean age of 48 ± 9 years. All patients had clinical and radiological assessments. The survival curves were calculated based on the following endpoints: unicompartmental or total knee arthroplasty.

**Results:**

The mean follow‐up was 15.2 ± 4.4 [10–29] years. The mean preoperative mechanical FemoroTibial Axis (mFTA) was 188.8° ± 3.2° [184°–197°], primarily due to femur deformity (mean lateral distal femoral axis [LDFA] 83.2° ± 2.8°). Bone union was achieved in 89.5% of patients (*n* = 34) at a mean delay of 6.5 ± 6.7 months. The complication rate was 26% (five stiffness, one nonunion, three secondary displacements and one deep vein thrombosis). Nine revision surgeries (24%) were recorded. Survival rates at 5 and 10 years were 92.1% and 78.9%, respectively. The mean delay between DFO and total knee arthroplasty (TKA) was 11.6 ± 5.7 [1–27] years. Nineteen patients (50%) were free of TKA at the last follow‐up. KSS scores were improved significantly. Return to sports was obtained in 92% of cases (*n* = 35), with a mean delay of 11 ± 8 months. Seventy‐four per cent of patients were satisfied or very satisfied with the surgery. Eighty‐four per cent would be willing to undergo the surgery again. Older age (*p* = 0.032) was a significant risk factor for TKA conversion.

**Conclusion:**

LOW‐DFO is an efficient procedure to manage lateral knee osteoarthritis in young patients with valgus deformity, with a good survival rate at 10 years and high patient satisfaction.

**Level of Evidence:**

Level III.

**Abbreviations**
BMI: body mass index
DFO: distal femoral osteotomy
HKA: hip knee ankle angle
KOOS: knee injury and osteoarthritis outcomes score
KSS: knee society score
LDFA: lateral distal femur angle
LOW‐DFO: lateral opening wedge distal femoral osteotomy
mFTA: mechanical FemoroTibial Axis
MPTA: medial proximal tibial angle
OA: osteoarthritis
TKA: total knee arthroplasty

## INTRODUCTION

Distal femoral osteotomy (DFO) is a knee‐preserving procedure for managing symptomatic lateral tibiofemoral osteoarthritis (OA) [20] in young, active patients with valgus deformity [10]. The biomechanical effect of limb alignment correction has been well described [29, 38]. DFO offers the opportunity to reduce knee pain, enhance the ability to return to higher‐level activities [3, 9] and delay the natural history of OA with the need for knee arthroplasty [5].

Several surgical techniques and approaches to perform DFO have been described. Most published studies of DFO have concerned medial closing wedge techniques, with fewer reports of lateral opening techniques, primarily due to the risk of nonunion. However, lateral opening wedge DFO (LOW‐DFO) is potentially easier to perform. An appropriate surgical technique allows more accurate angular correction, degree by degree, than a medial closing wedge osteotomy, and nonunion can be avoided using a bone graft [13]. Despite advantages and disadvantages, both lead to the same clinical outcomes, with a preference for the LOW‐DFO procedure [25, 37]. Clinical outcomes and survivorship of LOW‐DFO procedure have been described only in the short‐ and medium‐term cohorts, with small patient numbers [4, 8, 11, 18]. No long‐term evaluation exists in the literature.

This study aimed (1) to determine complications and survival rates of LOW‐DFO in the long‐term, (2) to assess their clinical outcomes (range of motion, knee society score (KSS), patient satisfaction, return to sports) in the long‐term and (3) to identify their risk factors of failure. The hypothesis was that LOW‐DFO has a high survival rate with good functional outcomes in the long term.

## MATERIALS AND METHODS

### Patients

From January 1991 to December 2011, 62 LOW‐DFO (60 patients) were performed in the same department. The inclusion criteria of this retrospective study were all patients who underwent an isolated LOW‐DFO for isolated lateral tibiofemoral OA and valgus malalignment, with a minimum follow‐up of 10 years. All grades of lateral femorotibial compartment preoperative OA were included, according to the Kellgren and Lawrence classification [17]. Exclusion criteria were as follows: DFO performed for instability (*n* = 6), associated procedures (such as tibial osteotomy, anterior cruciate ligament reconstruction, and tibial tuberosity osteotomy) (*n* = 6), revision of a previous DFO (*n* = 1), posttraumatic valgus deformity (*n* = 9) and a follow‐up less than 10 years (*n* = 2).

Thirty‐eight patients were included in the final analysis with a mean follow‐up of 15.2 ± 4.4 [10–29] years. All patients had a functional and radiological assessment at the most recent follow‐up. The mean age at surgery was 48 ± 9 [23–61] years. The mean preoperative mechanical FemoroTibial Axis (mFTA) was 188.8° ± 3.2° [184°–197°], primarily due to femoral deformity (mean lateral distal femoral axis [LDFA] 83.2° ± 2.8° [78°–88°]) (Table 1).

**Table 1:** Demographics data.

	LOW‐DFO^a^
	*n* = 38
Age at surgery (years)	47.6 ± 9.4
(mean ± SD) [minimum–maximum]	[23.5–61.4]
Sex	
Male	14 (27%)
Female	24 (63%)
BMI (kg/m^2^)	26.3 ± 4.5
(mean ± SD) [minimum–maximum]	[18.4–39.2]
Side	
Right	24 (63%)
Left	14 (27%)
ASA score	
1	32 (84%)
2	6 (16%)
3	0 (0%)
Previous knee surgery	16 (42%)
Kellgren and Lawrence stage	
Stage 1	5 (13%)
Stage 2	10 (26%)
Stage 3	12 (32%)
Stage 4	11 (29%)

*Note*: Per cent values are expressed as the nearest whole digit.

Abbreviations: ASA, American Society of Anesthesiologists; BMI, body mass index (kg/m2); LOW‐DFO, lateral opening wedge distal femoral osteotomy.

^a^ Data are presented as mean ± standard deviation [minimum–maximum] or number (proportion).

#### Outcomes assessment

All patients had standardized clinical and radiological assessments at 3 months, 12 months, 24 months, 5 years and 10 years after surgery, and at the most recent follow‐up. In the case of TKA conversion, the last pre‐TKA clinical, radiological, and time from surgery were used as the most recent assessment.

A complication was defined as a negative evolution of the medical care related to the DFO. Hardware removal was not considered a complication. Major complications were defined as those that have life‐threatening sequelae or endanger the viability of the limb involved, particularly the necessity of a new procedure on the osteotomy in this study. A revision was defined as any new surgery related to the primary DFO, except hardware removal without associated procedure.

Plate removals were performed systematically and were not counted in the revision rate. Failure was defined as re‐intervention requiring arthroplasty.

The radiographic assessment included anteroposterior, lateral, and patellar axial views and standing full long‐leg radiographs. All radiographs were performed in the same hospital. Mechanical femorotibial angle (mFTA), lateral distal femoral angle (LDFA), and medial proximal tibial angle (MPTA) [27] measurements were performed on full long‐leg X‐ray by an independent reviewer (PACS, Philips Easy Vision system). Corrections were considered good if the postoperative mFTA was between 178° and 180°. Bone healing was radiographically evaluated using a modification of the Staubli and Schröter method, measuring osteotomy gaps on the AP view [31]. Union was achieved if almost one‐third of the osteotomy surface was filled [31].

Clinical evaluation included range of motion, the KSS (knee and function), the subjective knee injury and OA outcomes score (KOOS), and the rate of return to sport. Patient satisfaction was evaluated according to a 5‐point Likert scoring scale at the most recent follow‐up (very satisfied, satisfied, neutral, unsatisfied, or very unsatisfied).

#### Surgical technique

Three senior knee surgeons performed all the surgeries using the same surgical technique. Surgical planning was performed preoperatively, with a target mFTA at 180°. A lateral subvastus approach was used. The open‐wedge osteotomy was performed by a lateral supra‐trochlear approach. The gap of the opening wedge was filled with autologous bone graft harvested from the anterior iliac crest (*n* = 34, 89%) or the ipsilateral tibial metaphysis (*n* = 4, 11%). All osteotomies were fixed using a 95° blade plate with cortical screws. Intraoperative fluoroscopy was utilized to measure mFTA correction before closure.

Postoperative rehabilitation protocol was standardized. Passive flexion and extension exercises were allowed. Patients had no weight bearing for 8 weeks. After clinical and radiological assessment, partial then full weight bearing was allowed from 8 weeks postoperatively.

#### Statistical analysis

Statistical analysis was performed with XLSTAT (2021, Addinsoft). Continuous variables were described using means, standard deviation, and ranges. Categorical variables were described using counts (per cent). Categorical outcomes were compared using Fisher's exact test and chi‐squared test. Normally distributed continuous variables were compared using Student's *t‐*test. The survival curves were calculated using the Kaplan–Meier method with a 95% confidence interval based on the following endpoints: unicompartmental or total knee arthroplasty. The logistic regression model to investigate risk factors of failures included classical demographic data and all the data that emerged as relevant from the univariate analysis. A *p*‐value < 0.05 was considered statistically significant for all analyses.

## RESULTS

The target for knee alignment was obtained in 29 procedures (76%). The mean correction on LDFA and mFTA was 8.8° ± 4.1° [4°–18°] and 9.7°± 4.3° [3°–18°], respectively, using the 3 months follow‐up long‐leg axis.

**Figure 1:**
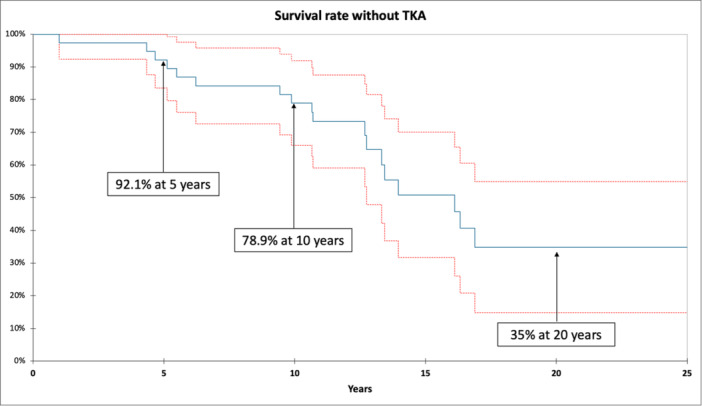
Ten‐year Kaplan–Meier survival estimate of lateral opening wedge distal femoral osteotomy without knee arthroplasty.

**Figure 2:**
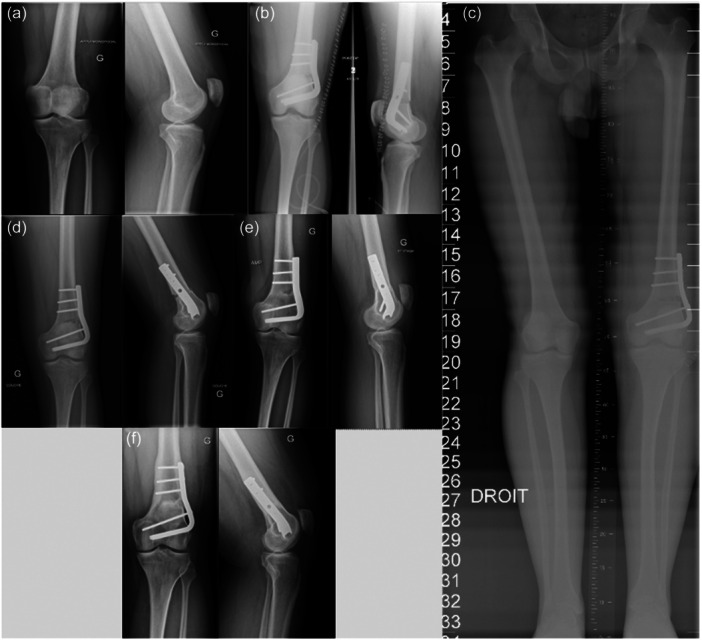
Case report of 45‐year‐old patient operated on lateral opening wedge distal femoral osteotomy (LOW‐DFO) for osteoarthritic knee on genu valgum. (a) Preoperative weight‐bearing knee X‐ray, with grade 2 lateral femorotibial compartment osteoarthritis. (b) Immediate postoperative (Day 0) X‐ray of the DFO, using a 95° blade plate with cortical screws. (c) Immediate postoperative (Day 3) long‐leg axis. Mechanical femorotibial angle was 176°, after a correction of 9°. (d) X‐ray at 2 months follow‐up, with a union of the DFO. (e) X‐ray at 1‐year follow‐up. (f) X‐ray at last follow‐up (11 years).

**Figure 3:**
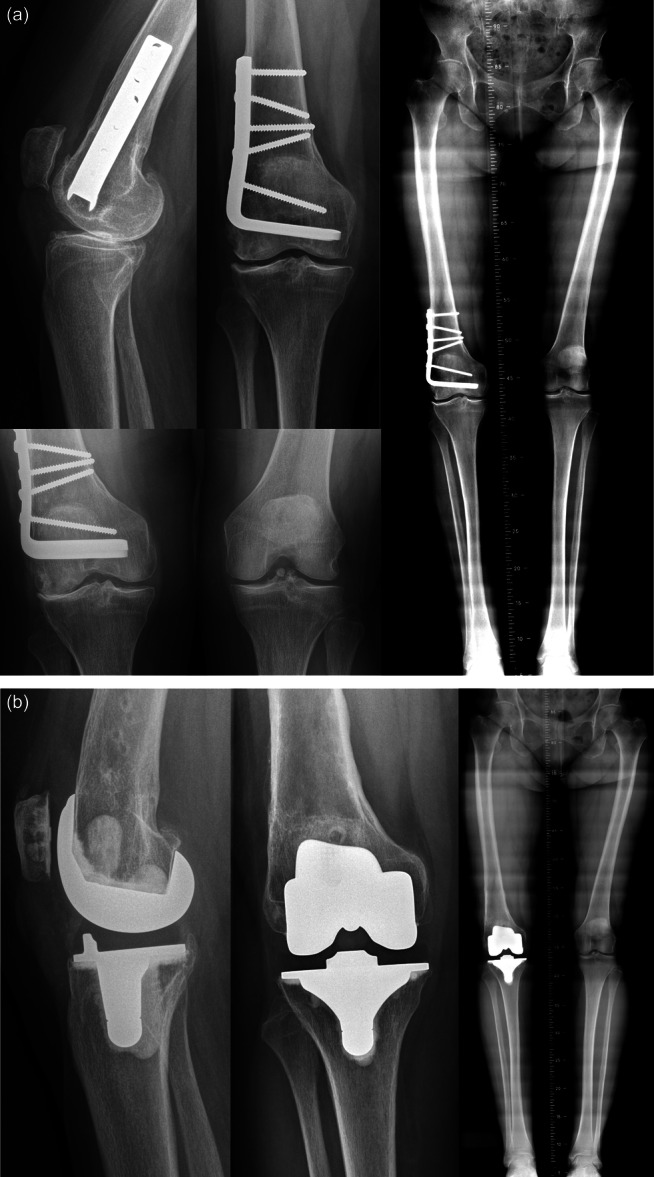
Case report of 56‐year‐old patient operated on lateral opening wedge distal femoral osteotomy (LOW‐DFO), with progression of lateral femorotibial osteoarthritis. (a) Radiographs at 16 years of follow‐up after the DFO showed lateral femorotibial osteoarthritis. (b) Radiographic control after the total knee arthroplasty performed at 16 years post‐DFO.

There were no intraoperative complications. Ten (26%) postoperative complications related to the DFO were recorded, with 9 (23.6%) requiring surgery (Table 2). The rate of major complications was 13.2% (5/38), including one deep vein thrombosis (2.6%), one nonunion (2.6%) and three secondary displacement of plate (7.9%). Bone union was achieved in 89.5% of patients (*n* = 34) at a mean delay of 6.5 ± 6.7 [2–36] months. Survival rate without knee arthroplasty was 92.1% at 5 years and 78.9% at 10 years (Figure 1). Nineteen patients (50%) were free of TKA conversion at the most recent follow‐up (Figure 2). The mean delay between DFO and TKA was 11.6 ± 5.7 [1–27] years. The main cause of conversion was the progression of lateral compartment OA (*n* = 9, 47%) and global OA (*n* = 9, 47%), at a mean delay of 12.5 ± 5.5 [5–27] years (Figure 3). One case required a conversion one year after DFO because of osteosynthesis failure. In the TKA conversion subgroup, the last assessment before conversion found a mean KSS knee score of 66.8 ± 14.7 [37–88] and a mean function score of 74.6 ± 21.8 [30–100].

**Table 2:** Complications and re‐operations related to distal femoral osteotomy (DFO).

	Number	Mean delay (months) (mean ± SD) [minimum–maximum]
Complications related to DFO	10 (26.3%)	8.3 ± 9.6 [1–28]
Minor complications	5 (13.2%)	13.0 ± 10.9 [3.5–28]
− Stiffness	5 (13.2%)	6
Major complications	5 (13.2%)	2.7 ± 2.7 [1–5]
− Nonunion	1 (2.6%)	1
− Secondary displacement of the plate	3 (7.9%)	
− Deep vein thrombosis	1 (2.6%)	
Re‐operations related to DFO	9 (23.6%)	9.2 ± 9.9 [1–28]
− Mobilization under anaesthesia	1 (2.6%)	3.5
− Arthroscopic arthrolysis	3 (7.9%)	18.7 ± 10.6 [7–28]
− Judet's procedure (extensive open arthrolysis)	1 (2.6%)	5
− Bone graft + new osteosynthesis	4 (10.5%)	3.5 ± 2.9 [1–6]

No significant difference was found between pre‐ and postoperative flexion range. KSS score was significantly improved post‐DFO (Table 3). In the group without TKA conversion, at the most recent follow‐up (14.1 ± 3.4 [10–20]), the KSS score was still improved compared to preoperative values (Table 3) with a mean KSS knee score of 80.5 ± 7.9 [67–89] and a mean function score of 85.5 ± 11.0 [65–100]. Return to sports was obtained in 92% of patients (*n* = 35), with a mean delay of 11.0 ± 7.9 [4–37] months. Thirty‐four of 35 (97%) patients returning to sport re‐obtained the same level as the preoperative period. Seventy‐four per cent of patients were satisfied or very satisfied with the surgery (*n* = 28). Eighty‐four per cent (*n* = 32) would be willing to undergo the surgery again, with full knowledge of the facts.

**Table 3:** Clinical outcomes of LWO‐DFO free of TKA conversion during follow‐up.

Delay	Preoperative	3 months post‐op	1‐year post‐op	2 years post‐op	Last follow‐up in the group without TKA conversion (*n* = 19)
Range of motion					
Recurvatum	3° ± 4	1° ± 2	2° ± 3	2° ± 4	1° ± 2
	[0–15]	[0–5]	[0–15]	[0–10]	[0–5]
Flexum	1° ± 2	1° ± 2	1° ± 2	0° ± 1	2° ± 3
	[0–10]	[0–5]	[0–5]	[0–3]	[0–5]
Full flexion	127° ± 23	112° ± 30	124° ± 22	124° ± 14	118° ± 12
	[30–150]	[5–145]	[60–150]	[100–140]	[100–130]
*p*‐Value	Ref	n.s.	n.s.	n.s.	n.s.
KSS score					
Knee	73.9 ± 12.6	81.7 ± 13.3	83.7 ± 14.6	87.0 ± 8.4	80.5 ± 7.9
	[40–95]	[37–97]	[25–96]	[66–100]	[67–89]
Function	80.9 ± 16.0	86.2 ± 18.3	91.7 ± 17.2	94.4 ± 13.9	85.5 ± 11.0
	[45–100]	[30–100]	[20–100]	[45–100]	[65–100]
Total	150.4 ± 27.1	167.6 ± 30.1	174.6 ± 31.5	179.6 ± 22.1	165.1 ± 14.8
	[100–195]	[67–195]	[45–196]	[111–200]	[133–188]
*p*‐Value	Ref	**0.019**	**0.0003**	**0.001**	0.25
KOOS					
Total	NR	NR	NR	NR	73 ± 15 [41–100]
Symptoms	NR	NR	NR	NR	74 ± 16 [43–100]
Pain	NR	NR	NR	NR	80 ± 14 [56–100]
Daily living	NR	NR	NR	NR	87 ± 12 [66–100]
Sports	NR	NR	NR	NR	56 ± 25 [0–100]
Quality of life	NR	NR	NR	NR	66 ± 26 [6–100]
mFTA (°)	188.8° ± 3.2	179.2° ± 3.3	NR	NR	NR
	[184–197]	[170–185]			
LDFA (°)	83.2° ± 2.8	92.0° ± 2.9	NR	NR	NR
	[78–88]	[88–99]			
MPTA (°)	90.1° ± 1.8	90.0° ± 2.0	NR	NR	NR
	[85–96]	[85–95]			

*Note*: Data are presented as mean ± standard deviation [minimum–maximum]. Bold values correspond to significant *p*‐value.

Abbreviations: KOOS, knee injury and osteoarthritis outcomes score; KSS, knee society score; LDFA, lateral distal femur angle; LOW DFO, lateral opening wedge distal femoral osteotomy; mFTA, mechanical FemoroTibial Axis; MPTA, medial proximal tibial angle; TKA, total knee arthroplasty.

Older patients had a higher risk of TKA conversion (Table 4). No other risk factor for procedure failure was identified (including demographic characteristics, preoperative clinical outcomes, previous knee surgery, complication or revision surgery due to DFO).

**Table 4:** Comparison of patients undergoing conversion to TKA and patients without TKA conversion.

	Conversion to TKA (*n* = 19)	Without TKA conversion (*n* = 19)	*p*‐Value
Age at surgery (years)	50.8 ± 6.4 [36–58]	44.4 ± 10.8 [23–61]	**0.032**
Follow‐up (years)	16.4 ± 5.1 [10–29]	14.1 ± 3.4 [10–20]	0.128
Sex (female)	7 (37%)	7 (37%)	0.737
BMI (kg/m^2^)	27.3 ± 5.2 [19–39]	25.3 ± 3.6 [18–32]	0.170
Tabaco	1 (7%)	4 (22%)	0.355
Previous knee surgery	9/17 (53%)	8/18 (44%)	0.164
ASA score			0.660
− ASA 1	17 (89%)	15 (79%)	
− ASA 2	2 (11%)	4 (21%)	
Preoperative osteoarthritis stage			
(Kellgren and Lawrence stage)			0.079
− 1	1 (6%)	4 (21%)	
− 2	2 (12%)	6 (32%)	
− 3	9 (53%)	3 (15%)	
− 4	5 (29%)	6 (32%)	
Preoperative flexion ROM (°)	126.5 ± 16.6 [90–150]	127.4 ± 27.5 [30–145]	0.165
Preoperative KSS score			
− Knee	74.7 ± 14.8 [40–95]	73.4 ± 11.3 [48–90]	0.777
− Function	83.7 ± 17.6 [50–100]	78.7 ± 14.7 [45–100]	0.250
− Total	148.4 ± 31.1 [100–195]	152.1 ± 24.2 [107–180]	0.702
Preoperative mechanical angle			
− mFTA (°)	189.6 ± 3.7 [186–197]	188.3 ± 3.1 [184–194]	0.521
− LDFA (°)	84.3 ± 2.8 [80–88]	82.4 ± 2.8 [78–88]	0.079
− MPTA (°)	89.7 ± 0.8 [88–91]	90.1 ± 2.0 [85–93]	0.325
Postoperative mechanical angle (2 months‐follow‐up)			
− mFTA (°)	179.9 ± 2.3 [175–184]	178.3 ± 3.6 [170–182]	0.207
− LDFA (°)	91.0 ± 1.8 [88–94]	92.9 ± 3.5 [88–99]	0.088
− MPTA (°)	89.3 ± 1.2 [88–92]	90.2 ± 2.1 [85–94]	0.109
Degree of mFTA Correction (°)	9.4 ± 4.3 [4–18]	9.9 ± 4.4 [3–17]	0.781
Postoperative complications	5 (26%)	5 (26%)	1
Revision surgery	3 (16%)	3 (16%)	1

*Note*: Data are presented as mean ± standard deviation [minimum–maximum] or number (proportion). Bold values correspond to significant *p*‐value.

Abbreviations: ASA, American Society of Anesthesiologists; BMI, body mass index (kg/m^2^); KSS, knee society score; LDFA, lateral distal femoral angle; mFTA; mechanical FemoroTibial Axis; MFTA, mechanical femorotibial angle; MPTA, medial proximal tibial angle; ROM, range of motion.

## DISCUSSION

The main finding of this study was good long‐term survivorship after LOW DFO, with a high satisfaction rate (80%).

The goal of a LOW‐DFO in osteoarthritic knee is to delay knee arthroplasty by 10 or 15 years in young and active patients. The survivorship in this study was satisfying, with a mean delay before knee arthroplasty of 12 years. The literature reports similar survivorship after DFO, from 79% at 5 years to 82% at 10 years [4, 8, 11]. The surgical alternative to DFO was an early TKA. However, TKA in young and active patients often had complex outcomes, with knee‐related dysfunction and dissatisfaction after surgery and lower functional outcomes primarily due to a higher rate of depression and complications (septic and aseptic loosening) [7, 21]. Moreover, the literature reports more complications, revisions, and re‐revision surgeries on revision total knee arthroplasty in young people [16, 26, 35]. For this reason, the long survivorship of DFO delaying arthroplasty encouraged this therapeutic strategy despite high grades of lateral OA. Moreover, satisfying outcomes and survivorship have been described for TKA after DFO. Recently, Gaillard et al. highlighted comparable mid‐term results for TKA post‐DFO and primary arthroplasty [12], with some precautions for gap balancing and axis correction [12, 32]. These findings were reinforced in recent systematic reviews [5, 23] and long‐term results [5]. Standard implants can be used post‐DFO without the need for specific stems or high constraints [19].

Moreover, the complications were mainly minor, without severe consequences for the patient. Indeed, no vascular, neurologic, or infectious complication was reported in this cohort. Few studies reported outcomes after LOW‐DFO, mainly in the short and mid‐term, with small patient numbers. Results were similar to the current study regarding complications, with the most common issues being stiffness and OA progression. The literature demonstrates a satisfying rate of bone healing, above 90% [18, 33]. In this study, the main major complication was an early secondary displacement of the DFO. This complication, rarely reported in the literature, was probably due to the fixation method. Indeed, the 95° blade plate can be difficult to position. Fixation was probably less secure than with modern locking plates. For this reason, the fixation method has been changed in our department in favour of locking plates. As highlighted by Peez et al., [28] the most important part beyond the fixation is maintaining an intact and continuous hinge to limit the secondary displacement. To prevent this hinge fracture, the literature proposes to use a biplanar procedure [39], aiming a hinge point at the level of distal to the proximal margin of the adductor tubercle [36]. This makes the hinge point very important in the surgical procedure.

The current study reported an improvement in KSS score even at a 15‐year follow‐up, with an increased score of up to 30 points at 2–3 years postoperatively, similar to the published literature [1, 14, 34]. Knee flexion was not improved post‐DFO in the present study, but this was not the aim of this knee preservation surgery. To improve flexion and avoid post‐operative stiffness, authors propose a rehabilitation protocol including a flexion up to 90° since D0 and limiting wearing the brace only to the first days to avoid pain. Patients had a satisfying rate of return to sports (92% in the current study), similar to the literature [2, 6, 30]. One of the most important findings of this study was the high patient satisfaction and their willingness to undergo the same surgery, if necessary, for more than 80% of patients. Patients were convinced of the benefits of this preservation surgery, witnessing its legitimacy and efficiency.

In the literature, the two main risk factors for conversion to TKA after DFO are age and medial compartment OA [15, 24]. While the present study confirmed older age as a risk factor, no evidence was found regarding medial compartment OA due to our exclusion criteria for performing this procedure. Any bicompartmental OA was excluded before analysis, but the progression of a diffused OA remains as one of the most common indications of conversion to TKA in this study. The degree of correction was not a risk factor for failure in this study. A recent biomechanical study showed that a 5° overcorrection restores near‐normal contact pressure and contact area in the lateral compartment [29]. According to Quirno et al., [29] overcorrecting a few degrees could increase the survival rate and the delay before conversion, but this factor is still debated [22]. Indeed, the overcorrection could lead to other complications, including contralateral compartment degradation, joint line obliquity, and difficulties in conversion to TKA [12].

This study has several limitations. First, it was a retrospective study. Nevertheless, the main aim was the assessment of complications and survivorship, easily recorded retrospectively. The database was collected prospectively, and the minimum 10‐year follow‐up ensures that the majority of complications and revisions relating to the DFO will have been identified. Second, our study had a relatively small sample size. These limitations are inherent to this type of procedure, which has rare indications but should be considered when interpreting our results.

The main strength of the present study was the long‐term follow‐up in a monocentric cohort with the same surgical technique. Benefits from DFO, in well‐selected cases, were delaying arthroplasty with good functional outcomes and a high satisfaction rate [4, 6, 18]. Therefore, DFO was not only a delaying procedure but also a complete and efficient procedure for these osteoarthritic knees and should be part of the OA treatment strategy in an active and young population with valgus deformity.

## CONCLUSION

LOW‐DFO delayed TKA by a mean of 12 years. LOW‐DFO was an efficient procedure to manage lateral knee OA in young and active patients with valgus deformity, with a good survival rate at 10 years and high patient satisfaction.

## AUTHOR CONTRIBUTIONS

**Nicolas Cance**: Study design, data collection, literature review and manuscript writing. **Cécile Batailler**: Study design, statistical analysis, literature review, manuscript editing and supervision. **Timothy Lording**: Study design and manuscript editing. **Axel Schmidt**: Study design and statistical analysis. **Sébastien Lustig**: Study design and manuscript editing. **Elvire Servien**: Study design, supervision, and manuscript editing. All authors read and approved the final manuscript.

## CONFLICT OF INTEREST STATEMENT

Timothy Lording: Consultant for Amplitude, Speakers Bureau for Smith and Nephew and Arthrex. Sébastien Lustig: Consultant for Stryker, Smith Nephew, Heraeus, DePuy Synthes; Institutional research support from Groupe Lepine, Amplitude; Editorial Board for *Journal of Bone and Joint Surgery (Am)*. Elvire Servien: Consultant Smith and Nephew. The remaining authors declare no conflict of interest.

## ETHICS STATEMENT

All procedures were performed in accordance with the ethical standards of the institutional and/or national research committee, the 1964 Helsinki Declaration and its later amendments, or comparable ethical standards. Data collection and analysis were carried out in accordance with MR004 Reference Methodology from the Commission Nationale de l'Informatique et des Libertés (Ref. 2229975V0) obtained on 6 May 2023. The study was registered and filed on the Health Data Hub website.

## Data Availability

The data that support the findings of this study are not openly available.
